# How do Snow Partridge (*Lerwa lerwa*) and Tibetan Snowcock (*Tetraogallus tibetanus*) coexist in sympatry under high‐elevation conditions on the Qinghai–Tibetan Plateau?

**DOI:** 10.1002/ece3.8424

**Published:** 2021-12-08

**Authors:** Hongyan Yao, Pengcheng Wang, Geoffrey Davison, Yong Wang, Philip J. K. McGowan, Nan Wang, Jiliang Xu

**Affiliations:** ^1^ School of Ecology and Nature Conservation Beijing Forestry University Beijing China; ^2^ Key Laboratory of Animal Ecology and Conservation Biology Institute of Zoology Chinese Academy of Sciences Beijing China; ^3^ National Biodiversity Centre National Parks Board Singapore City Singapore; ^4^ Department of Biological and Environment Sciences Alabama Agricultural and Mechanical University Huntsville Alabama USA; ^5^ School of Natural and Environmental Sciences Newcastle University Newcastle UK

**Keywords:** coexistence, high elevation, *Lerwa lerwa*, Qinghai–Tibetan Plateau, sympatry, *Tetraogallus tibetanus*

## Abstract

The Qinghai–Tibetan Plateau (QTP) has the highest elevations of all biodiversity hotspots. Difficulties involved in fieldwork at high elevations cause challenges in researching mechanisms facilitating species coexistence. Herein, we investigated Snow Partridge (*Lerwa lerwa*) and Tibetan Snowcock (*Tetraogallus tibetanus*), the only two endemic Galliformes on the QTP, to understand species coexistence patterns and determine how they live in sympatry for the first time. We assembled occurrence data, estimated habitat suitability differences and the underlying factors between two species at different scales using ecological niche models. Niche overlap tests were used to investigate whether niche differences between these species allow for their coexistence. We found that elevation was the most important factor determining habitat suitability for both species. At the meso‐scale, two species have similar ecological niches with their suitable habitats lying predominantly along ridge crests. However, ridge crests were more influential for habitat suitability by *L*. *lerwa* than for that of *T*. *tibetanus* because the latter species ranges further afield than ridge crests. Thus, differences in habitat suitability between these species lead to habitat partitioning, which allows stable coexistence. At the macro‐scale, temperature and precipitation were major factors influencing habitat suitability differences between these species. *Tetraogallus tibetanus* extended into the hinterland of the QTP and occurred at higher elevations, where colder and drier alpine conditions are commonplace. Conversely, *L*. *lerwa* occurred along the southeastern margin of the QTP with a lower snow line, an area prone to rainy and humid habitats. Niche overlap analysis showed that habitat suitability differences between these species are not driven by niche differentiation. We concluded that the coexistence of these two pheasants under high‐elevation conditions could be an adaption to different alpine conditions.

## Graphical abstract

We used Snow Partridge (*Lerwa lerwa*) and Tibetan Snowcock (*Tetraogallus tibetanus*), the only two endemic Galliformes on the Qinghai–Tibetan Plateau (QTP), to understand their range overlap and how they have involved differently to a mostly high‐elevation environment. *T. tibetanus* extended into the hinterland of the QTP and occurred at higher elevations, the species is adapted to a broader temperature range and drier conditions, while *L. lerwa* is adapted to southern and eastern edge of the QTP with a lower snow line, an area prone to rainy and humid habitats. The coexistence of these two pheasants under high‐elevation conditions could be an adaption to different alpine conditions.

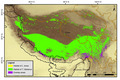

## 1 INTRODUCTION

The mechanisms facilitating the maintenance of biodiversity are important for research in ecology and biogeography (Myers et al., 2000), although their investigation may be difficult in high‐elevation areas. Among the biodiversity global hotspots, the Qinghai–Tibetan Plateau (QTP) show the highest elevation with an average elevation of 4500 m (Myers et al., 2000; Zhang et al., 2017) that impose challenging ecological, physical, and climatic conditions (Xu et al., 2009; Zemp et al., 2019). The QTP contains many taxonomically isolated and ecologically unique species (Jia, 2012; Lei & Lu, 2006). However, its biodiversity knowledge is far from complete since difficulties in conducting fieldwork delay the understanding of ecological and evolutionary processes of QTP species (Cai et al., 2018; Luo et al., 2019; Wu et al., 2013, 2014; Yao et al., 2017). The outstanding biodiversity of QTP in concert with its unique environment makes this hotspot ideal for researching species coexistence in mountainous regions. Previous studies investigating the QTP region have shown various examples of sympatric range via ecological separation in birds and mammals through mechanisms such as habitat partitioning, a partition of the environment in ways that minimizes interspecific competition (Bhatnagar et al., 2000; Byrne et al., 2019; Cui et al., 2008; Namgail et al., 2004; Wei et al., 1999). Although several studies have performed ecology‐related analyses of biodiversity at a broad scale across the QTP (e.g., Ding et al., 2006; Xu et al., 2017), to our knowledge, there remains insufficient research to explain the mechanisms facilitating species coexistence in high‐elevation areas (Fjeldså & Rahbek, 2006; Jetz & Rahbek, 2002; Rahbek et al., 2007).

The sympatric range of some species with similar ecological requirements can be related to local‐level habitat partitioning due to competition (i.e., habitat partitioning hypothesis) (Cui et al., 2008; Namgail et al., 2004). Stable coexistence requires there to be distinct ecological differences between species, and this usually involves niche differentiation (i.e., organisms coexist by partitioning available resources, such as food or space) and trade‐offs in resource use (Chesson, 2000). Generally, biotic and abiotic factors (e.g., competition and the physical environment) drive these types of interaction (Chesson, 2000; Gutiérrez et al., 2014). Habitat suitability—the ability of a habitat to support a viable population over an ecological time scale (Kellner et al., 1992)—can help us to understand the adaptive significance of habitat use for an available resource (Jones, 2001). Assessing the differences in species niches and/or habitat requires the identification and consideration of the underlying factors, including abiotic and biotic interactions, which influence species range limits (Guisan & Thuiller, 2005). Quantifying how environment affects biotic interactions within species geographic range is often difficult, and a more convenient strategy relies on the identification of which (if any) environmental factors underlie potential differences in the ecological niche of coexisting species. Within this context, ecological niche models (ENMs) have been used to compare how abiotic niches overlap between species (Broennimann et al., 2012; Hirzel & Le Lay, 2017; Warren et al., 2008). In comparing how niche overlap related to habitat suitability, we can better understand the role of environment in shaping species coexistence patterns.

The QTP harbors 29 species of Galliformes (Zheng, 2018), but Snow Partridge (*Lerwa lerwa*) and Tibetan Snowcock (*Tetraogallus tibetanus*) are the only two endemic Galliformes living in high‐elevation habitats of the QTP (Yao et al., 2017; Zheng, 2016). *Lerwa lerwa* is distributed along the southeastern margin of the plateau at elevations ranging from 3,800 m to 5,200 m near the permanent snow line (Cheng et al., 1978; del Hoyo et al., 1994; Yao et al., 2017). *Tetraogallus tibetanus* extends into the hinterland of the plateau and ranges from 3700 m up to 6000 m, above the snow line (Cheng et al., 1978). These two species occur sympatrically through much of their habitat (Cheng et al., 1978; Madge & McGowan, 2002), and it has been suggested that niche similarity may lead to competition between *L*. *lerwa* and *T*. *tibetanus* (Li & Lu, 1992). The seemingly similar ecological niches of these species provide an opportunity to study the coexistence mechanisms facilitating their sympatric range on the QTP. Additionally, with glacial retreat due to climate change documented from 2006 to 2016, these two species face the upward movement of the snow line (Xu et al., 2009; Zemp et al., 2019). This is particularly true for *L*. *lerwa* whose geographic range along the southeastern margin of the plateau is more closely associated with the snow line than that of *T*. *tibetanus*, which reaches arid parts of the hinterland of the plateau where snow is uncommon.

To our knowledge, the present study is the first to explore the mechanisms of species coexistence at elevations as high as those on the QTP. To achieve this, we compared the differences in habitat suitability and the underling factors between these two sympatric high‐elevation pheasants specifically in terms of resource use and topographic and climatic factors at different scales. We further used niche overlap analysis to test whether niche differentiation as a consequence of evolutionary adaptation (i.e., the hereditary alteration or adjustment in structure or habits of a species or individual to survive) resulted in the niche differences of *L*. *lerwa* and *T*. *tibetanus* to better understand their coexistence. Habitat partitioning including topographical and vegetation factors has explained how two pheasants are able to maintain sympatric range (Cui et al., 2008). *Lerwa lerwa* is distributed along the southeastern margin of the QTP; *Tetraogallus tibetanus* extends into the hinterland of the plateau with larger elevation ranges than *L*. *lerwa*. Alternatively, the most prominent topographic structure of the QTP is characterized by a flat interior surrounded by high montane ranges (Lei et al., 2014) and the central platform of the QTP is cold and arid year‐round (Hamerlík & Jacobsen, 2012; Xu et al., 2009). Thus, we hypothesized that habitat partitioning related to elevation, climatic conditions (e.g., temperature and precipitation) would determine the coexistence of these two high‐elevation species.

## 2 MATERIALS AND METHODS

### 2.1 Field sampling protocol

We surveyed suitable habitats repeatedly in the Balangshan mountains (30°53ʹ–30°57ʹN, 102°52ʹ–102°54ʹE) within the administrative counties of Xiaojin, Wenchuan, and Baoxing (these three counties are hereafter referred to as the meso‐scale) in August 2013, which are at the southeastern margin of the QTP (Figure S1 in Appendix S2). We used a line transect method and playbacks. Playbacks are an effective method for increasing the detection of Galliformes (Wang et al., 2004). In the mornings when *L*. *lerwa* leave their roosting sites and in the evenings when they returned, these birds can commonly be heard communicating as a group. In addition to calling when a perceived predator, such as Red Fox (*Vulpes vulpes*), or large herbivores, such as Domestic Yak (*Bos grunniens*) and Blue Sheep (*Pseudois nayaur*), are in close proximity, *L*. *lerwa* responded to playbacks in the morning. On rainy and foggy days, *L*. *lerwa* called periodically during the daytime. The calls revealed the position of the birds, and the open habitat allowed us to approach and locate them precisely from within distances of up to 600 m. We identified the position of the roosting site of each flock by listening before sunrise (approximately 05:45 h) along the road and paths in the area. During the daytime surveys, we walked at a speed of 1.5‒2.5 km/h to cover the range of elevations of a specific ecological or plant community between 4000 and 4800 m. Once a *L*. *lerwa* flock was detected, we stopped and maintained a distance of >100 m to observe the movements of the flock. We assumed that this procedure was adequate to minimize observer effects given that these birds are protected. There was no evidence of hunting within the study area, and *L*. *lerwa* are often undisturbed by people passing within 50 m of their position (Yao et al., 2017). We determined the location of birds using a hand‐held GPS receiver (Garmin GPS12C) once the flock had left, or alternatively, we plotted the location on the study area map if the area was too steep to immediately follow.

The Balangshan mountains contained habitats used by both *L*. *lerwa* and *T*. *tibetanus* in which these two species occurred in mixed groups and were observed in the same foraging habitats and roosting sites during the post‐breeding period. Considering that the Balangshan mountains contain typical sympatric ranges of these two species, we used the occurrence records from these mountains to compare differences in habitat suitability at the meso‐scale. The field surveys of *T*. *tibetanus* were conducted synchronously with those of *L*. *lerwa* using the same survey method.

### 2.2 Compilation of species occurrence records

At the meso‐scale, 71 occurrences of *L*. *lerwa* and 42 occurrences of *T*. *tibetanus* were obtained during fieldwork in the Balangshan mountains, concentrated within 12 km^2^ of mountain slopes over a period of 20 days (approximately 320 h) in the 526‐km^2^ area (Figure S1). Occurrence points separated by at least 30 m were considered distinct (Yao et al., 2017) and were used to construct the model for subsequent comparison of habitat suitability.

At the macro‐scale of the QTP, species occurrences were recorded from field surveys, published references (see Appendix S1), and an online database (Global Biodiversity Information Facility, GBIF; http://www.gbif.org/), eBird (http://ebird.org/) and xeno‐canto (http://www.xeno‐canto.org). We conducted another field survey in the Shangri‐la (28°9ʹ–28°16ʹN, 99°54ʹ–99°58ʹE) in January 2015 and subsequently in the Yulong mountains (27°14ʹ–27°3ʹN, 100°8ʹ–100°13ʹE) in August 2016 both in Yunnan Province, China (Figure S1), to record occurrence. We removed duplicates and occurrences within 1‐km distance since all occurrences nearly covered the whole geographic range of each species and met 1‐km fine resolution. Finally, 104 occurrences of *L*. *lerwa* and 328 occurrences of *T*. *tibetanus* were used for habitat suitability prediction (Figure S1).

### 2.3 Environmental predictors

At the meso‐scale, we scored environmental variables that were potentially important for *T*. *tibetanus* and *L*. *lerwa* (elevation, slope, aspect, distance to the nearest ridge crest, and vegetation) (see Appendix S1; Table S1 in Appendix S3).

At the macro‐scale, we used 19 bioclimatic factors and one elevation factor with a spatial resolution of 30 arc‐seconds (~1km) from the climate data available on WorldClim v.2 (http://worldclim.org/) to construct ENMs to predict the potential habitat suitability of each species. Among the 20 predictors, strong multicollinearity could result in overfitting the ENMs. We have considered using variance inflation factor (VIF) to detect multicollinearity. However, remained variables by VIF analysis contained eight climate–energy variables without elevation (Appendix S1). This study aims to explore how two pheasants coexist under high‐elevation conditions in the QTP. Elevation as a potential important factor would be better remained. Besides, species richness usually is determined by multi‐factors such as climate energy and habitat heterogeneity (Jimenez‐Alfaro et al., 2016; Moura et al., 2016). Finally, we used Pearson's correlations to detect collinearity between every two predictors and the remained variables met our aim (details see Appendix S1). A pair of variables with Pearson's correlation > |0.80| were regarded as highly correlated and which is more important in biological and ecological meaning for species was remained (Fouquet et al., 2010; Table S2 in Appendix S3). Hence, eight variables were selected: elevation (Ele, m), mean diurnal range (mean of monthly (max temp −min temp), MDR, °C), temperature seasonality (TS, standard deviation *100), mean temperature of driest quarter (MTDQ, °C), annual precipitation (AP, mm), precipitation in driest month (PDM, mm), precipitation seasonality (PS, coefficient of variation), and precipitation in coldest quarter (PCQ, mm).

### 2.4 Ecological niche modeling

A model algorithm of Maxent 3.3.3e (Phillips et al., 2006) was used to construct ENMs to predict the potential habitat suitability of species at the meso‐scale and macro‐scale. Maxent performance is based on presence‐only data (Elith et al., 2006). Of all locations, 75% were selected as a training set, and the remaining 25% were used to randomly test the models. A logistic output was selected to represent the logistic habitat suitability values ranging from 0 to 1. The recommended default parameters are given in parentheses for the convergence threshold (10^−5^), regularization multiplier (1), bootstrap, the maximum number of iterations (500), and 10 replicates. The area under the receiver operating characteristic curve (AUC) was used to assess predictive performance. Jackknife test, a statistical resampling method used to derive an estimate of bias and standard error of the variance of the sample (McIntosh, 2016), was used to estimate the apparent importance of variables (i.e., rank of variable contribution to predictions relatively) in habitat suitability predictions, with higher AUC values indicating more important variables (Yao et al., 2017). For the logistic output of the ENMs of *L*. *lerwa* and *T*. *tibetanus*, a binary transformation was used to obtain the potential suitable habitat based on the 10th percentile training presence logistic threshold for each species. Under this threshold, these two species had appropriate elevation ranges as per a previous study (Yao et al., 2017).

### 2.5 Data analysis

To test the habitat partitioning hypothesis, we compared potential habitat suitability areas and the underlying factors of *L*. *lerwa* and *T*. *tibetanus*. We overlaid the habitat suitability areas between *L*. *lerwa* and *T*. *tibetanus* using spatial analysis tools with “Extract by mask” in ArcGIS 10.2 (Tang & Yang, 2006). To test the habitat partitioning hypothesis related to differences in environmental factors, the random forest modeling technique was used to rank the importance of the environmental variables for distinguishing the potential habitat suitability between *L*. *lerwa* and *T*. *tibetanus* at the macro‐scale with the “randomForest” package in R (Liaw & Wiener, 2015; R Core Team, 2019). The random forest approach uses a combination of decision tree predictors so that each tree depends on the values of a random vector sampled independently with the same distribution applied to all trees in the forest (Breiman, 2001). The two indicators, the “mean decrease accuracy” and the “mean decrease Gini,” both refer to the importance of variables; for both indicators, the greater the value, the more important the variable. Information for eight environmental variables of occurrences of *L*. *lerwa* and *T*. *tibetanus* was extracted from the data layers in ArcGIS 10.2. The chi‐squared test was used to test the differences in important environmental variables between *L*. *lerwa* and *T*. *tibetanus* at the macro‐scale. When the data conformed to normal distribution, an independent‐sample *t* test was used; otherwise, a Mann–Whitney *U* test was used (Wang et al., 2005). Results are reported as mean ± SE.

To test whether the two species have similar ecological or climate niches, we used the updated niche overlap tests to assess the niche overlap of *L*. *lerwa* and *T*. *tibetanus* (Broennimann et al., 2012). This new method applied a kernel smoother to densities of species occurrence in environmental space to calculate metrics of niche overlap and test hypotheses regarding niche conservatism. This is a robust method for quantifying niche differences between species (Broennimann et al., 2012). Niche overlap was statistically tested using niche equivalency and similarity tests (Warren et al., 2008). The niche equivalency test determines whether niches of two species in two geographical ranges are equivalent when randomly reallocating the occurrences of both species among the two ranges (Broennimann et al., 2012). We used the niche similarity test to determine whether the niches of the two species were more similar than expected by chance (Broennimann et al., 2012; Warren et al., 2010). The niche similarity test differs from the equivalency test because the former examines whether the overlap between observed niches in two ranges is different from the overlap between the observed niche in one range and niches selected at random from the other range. Schoener's *D* (Schoener, 1968) and Hellinger's *I* (Warren et al., 2008) metrics ranging from 0 (no overlap) to 1 (complete overlap) were used to measure niche overlap, and the equivalency and similarity. The null hypothesis of the niche similarity test is that overlaps in the range of two paired species are by chance, which is rejected if the *D* and *I* values are not within the 95% confidence limits. *D* and *I* values to the right of the 95% confidence limits indicate that the ecological niches are conserved, whereas values to the left indicate that the ecological niches are divergent (Warren et al., 2008). A null distribution from 1000 pseudo‐replicates was obtained by pooling and randomizing the occurrence points throughout the study regions of both species at two different scales. At the meso‐scale, the geographic range was set with a 1.02‐km buffer for occurrences (Yao et al., 2017). At the macro‐scale, the geographic range was set for a 200‐km buffer (Lyu et al., 2015). The analyses were conducted in R 3.6.1 with the package “ecospat” (Di Cola et al., 2017).

## 3 RESULTS

**FIGURE 1:**
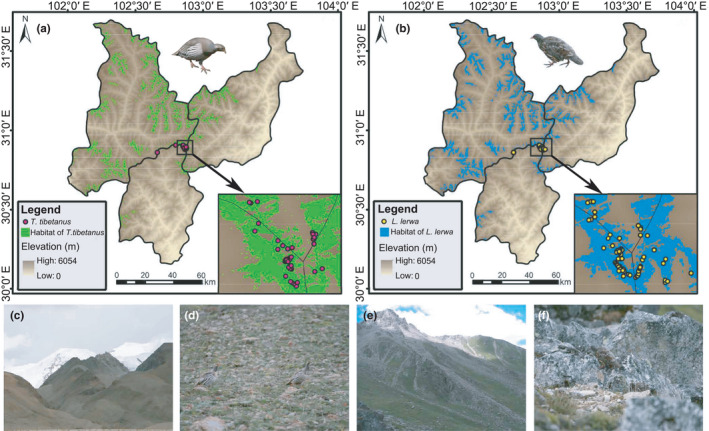
Habitat suitability areas for *Tetraogallus tibetanus* and *Lerwa lerwa* in the Balangshan mountains on the eastern Qinghai–Tibetan Plateau (QTP) within Xiaojin, Wenchuan, and Baoxing counties, Sichuan Province, China, by ecological niche models, and typical habitats and species on the QTP: (a) habitat suitability areas for *T*. *tibetanus*, (b) habitat suitability areas for *L*. *lerwa*, (c) typical habitats used by *T*. *tibetanus* in Pulan county of the Ali ranges of Tibet, (d) photograph of *T*. *tibetanus* in Pulan, (e) typical habitats used by *L*. *Lerwa* in the Balangshan mountains, and (f) photograph of *L*. *lerwa* in Cuona county, Tibet. Photographs c‐f were all taken by Nan Wang during filed surveys

**FIGURE 2:**
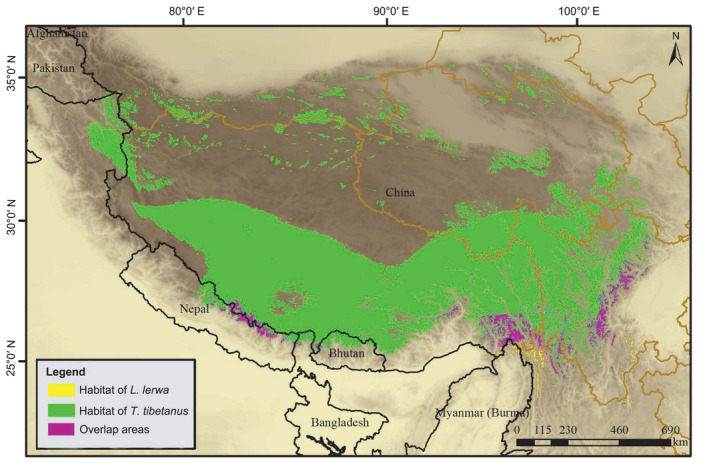
Habitat suitability predictions of *Lerwa lerwa* and *Tetraogallus tibetanus* across the Qinghai–Tibetan Plateau, including the 47,890‐km^2^ overlapping area. The overlapping area constituted approximately 88.92% and 5.28% of the habitat suitability of *L*. *lerwa* and *T*. *tibetanus*, respectively

The high AUC values of the model indicated that the potential habitat suitability was predicted accurately (Table S3 in Appendix S3). At the meso‐scale, the habitat suitability area for *T*. *tibetanus* and *L*. *lerwa* occupied 7.47% and 6.64% of the entire area, respectively (Figure 1a,b; Table S3). The habitats for both species were concentrated along the ridge crests and that of *T*. *tibetanus* was slightly more extensive than that of *L*. *lerwa*. However, the abundance of *T*. *tibetanus* at 41 was lower than that of *L*. *lerwa* at 108 according to our surveys. At the macro‐scale, the areas of overlap between these two pheasants totaled 47,890 km^2^, occupying approximately 88.92% and 5.28% of the potential habitat suitability of *L*. *lerwa* and *T*. *tibetanus*, respectively (Table S3). This result indicated a large difference between the potential habitat suitability of these two species. *Lerwa lerwa* are limited to the southeastern margin of the QTP, while *T*. *tibetanus* extended into the hinterland of the plateau (Figure 2).

**FIGURE 3:**
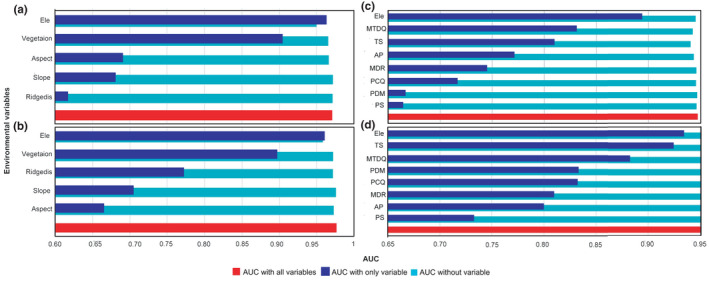
Jackknife test of the importance of environmental variables in habitat suitability areas for *Tetraogallus tibetanus* and *Lerwa lerwa* at two different scales on the Qinghai–Tibetan Plateau: (a) *T*. *tibetanus* at the meso‐scale, (b) *L*. *lerwa* at the meso‐scale, (c) *T*. *tibetanus* at the macro‐scale, and (d) *L*. *lerwa* at the macro‐scale. The larger the AUC value, the more important the variable. AP, annual precipitation (mm); Ele, elevation (m); MDR, mean diurnal range (mean of monthly (max temp −min temp), ℃); MTDQ, mean temperature of driest quarter (℃); PCQ, precipitation in coldest quarter (mm); PDM, precipitation in driest month (mm); PS, precipitation seasonality (coefficient of variation); Ridgedis, distance to ridge line; TS, temperature seasonality (standard deviation *100)

We found that elevation, vegetation, aspect, and slope were the most important environmental determinants of habitat suitability for *T*. *tibetanus* (Figure 3a). For *L*. *lerwa*, elevation, vegetation, distance to ridge crests, and slope mainly influenced habitat suitability (Figure 3b). Topographical factors along a ridge crest varied the most between the habitats selected by these two pheasants, which was the least influential of the five environmental factors for determining the habitat suitability of *T*. *tibetanus*. However, this was the most influential variable for *L*. *lerwa*. At the macro‐scale, the habitat suitability of *T*. *tibetanus* was mainly influenced by elevation, mean temperature of driest quarter, and temperature seasonality (Figure 3c). For *L*. *lerwa*, elevation was the most important environmental variable, followed by temperature seasonality, mean temperature of driest quarter, precipitation in driest month, precipitation in coldest quarter, mean diurnal range, and annual precipitation (Figure 3d). We found that the habitat suitability of *L*. *lerwa* was influenced by many more factors than that of *T*. *tibetanus*, particularly factors related to precipitation. In contrast, factors related to temperature variation were more influential for determining the habitat suitability of *T*. *tibetanus*.

**FIGURE 4:**
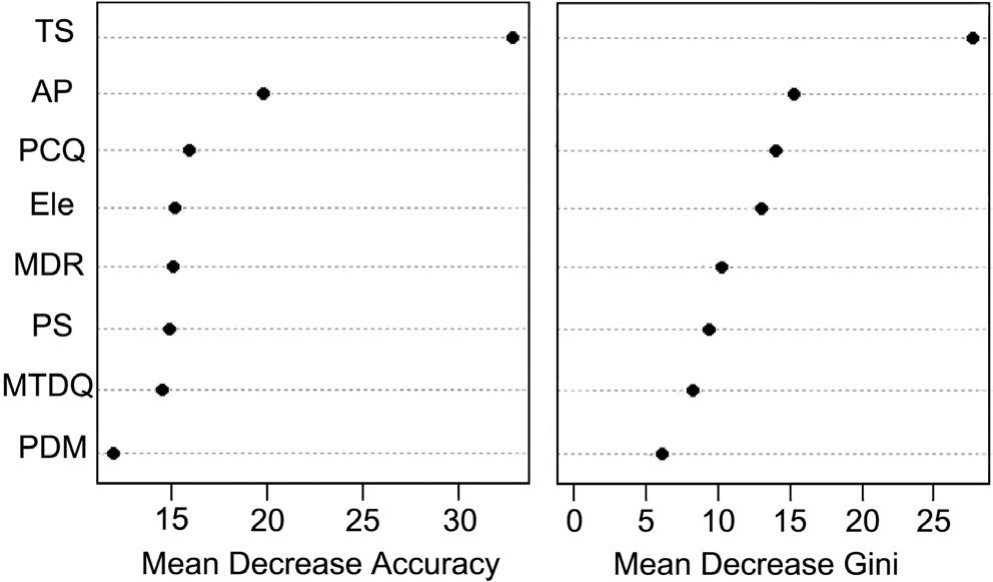
Importance ranking of environmental variables based on random forest analysis using the “mean decrease accuracy” or “mean decrease gini” method for habitat suitability comparison between *Lerwa lerwa* and *Tetraogallus tibetanus* on the Qinghai–Tibetan Plateau. The greater the value, the more important the variable. AP, annual precipitation (mm); Ele, elevation (m); MDR, mean diurnal range (mean of monthly (max temp −min temp), ℃); MTDQ, mean temperature of driest quarter (℃); PCQ, precipitation in coldest quarter (mm); PDM, precipitation in driest month (mm); PS, precipitation seasonality (coefficient of variation); TS, temperature seasonality (standard deviation *100)

Random forest results showed that seasonal temperature was the most influential variable affecting habitat suitability differences between the two species, followed by annual precipitation, precipitation in coldest quarter, and so on (Figure 4). We found significant differences in the eight environmental variables (*p* < .01; Table 1). The variables related to temperature variation (bio 2, bio 4, and bio 9) were significantly larger for *T*. *tibetanus* than for *L*. *lerwa* while the variables related to precipitation (bio 12, bio 14, and bio 19) were significantly larger for *L*. *lerwa* than for *T*. *tibetanus* (*p* < .01). Besides, diurnal and seasonal temperature, and precipitation seasonality varied more significant in the habitats of *T*. *tibetanus* than those of *L*. *lerwa* (*p* < .01), suggesting *L*. *lerwa* lived in more stable conditions than *T*. *tibetanus*.

**TABLE 1:** Average environmental conditions found within occurrences of *Lerwa lerwa* and *Tetraogallus tibetanus* on the Qinghai–Tibetan Plateau

Environmental variable	*Lerwa lerwa* (*n* = 104)	*Tetraogallus tibetanus* (*n* = 328)	*Z* ^a^	*T* ^b^	*p*
Ele (m)	4420.49 ± 379.10	4707.10 ± 543.64		−6.49	.000**
MDR (°C)	121.75 ± 9.50	136.93 ± 10.28	−10.86		.000**
TS	5462.19 ± 491.69	7019.38 ± 1183.37	−4.02		.000**
MTDQ (°C )	−59.57 ± 26.66	−98.23 ± 46.39	−11.79		.000**
AP (mm)	757.14 ± 249.04	413.28 ± 249.02	−10.32		.000**
PDM (mm)	5.24 ± 2.77	2.43 ± 2.01	−9.34		.000**
PS	93.38 ± 12.82	101.94 ± 19.78	−6.10		.000**
PCQ (mm)	27.38 ± 19.88	16.16 ± 16.60	−9.48		.000**

Abbreviations: AP, annual precipitation; Ele, elevation; MDR, mean diurnal range (mean of monthly (max temp −min temp)); MTDQ, mean temperature of driest quarter; PCQ, precipitation in coldest quarter; PDM, precipitation in driest month; PS, precipitation seasonality (coefficient of variation); TS, temperature seasonality (standard deviation *100).

*p < .05; **p < .01.

^a^ Mann–Whitney *U* test, *Z* value.

^b^ Independent‐samples *t* test, *t* value.

At the meso‐scale, the niche similarity tests showed that the ecological niches of *L*. *lerwa* and *T*. *tibetanus* were significantly similar (*D* = 0.78, *I* = 0.86; both *p* = .001 for *L. lerwa* as background, *p* = .026 and *p* = .007 for *T*. *tibetanus* as background; Figure S2), and both values in the two‐tailed test rejected the null hypothesis. These results show that the niches of these two species were conserved (or not differentiated) at the meso‐scale. At the macro‐scale, the ecological niches were also significantly similar when the range of *T*. *tibetanus* was used for the background test with *D* and *I* to the right of the 95% confidence interval (*D* = 0.48, *I* = 0.68; both *p* = .000; Figure S3). However, these were not significant when we considered the range of *L*. *lerwa* as the background, which does not indicate dissimilar niches let alone niche differentiation. At the two scales, however, the equivalency tests showed similar niches between *L*. *lerwa* and *T*. *tibetanus*, and these results were significant (*p* < .05, Figures S4 and S5). Therefore, the differences in habitat suitability between *L*. *lerwa* and *T*. *tibetanus* at the macro‐scale were not caused by niche differentiation over evolutionary time.

## 4 DISCUSSION

To our knowledge, ours is the first study to reveal the topographical (i.e., elevation and ridge crests) and climatic (i.e., temperature and precipitation) factors that influence the coexistence of *L*. *lerwa* and *T*. *tibetanus* in high‐elevation areas at the meso‐scale and macro‐scale on the QTP. Our results confirmed our hypothesis that habitat partitioning related to elevation, temperature, and precipitation would determine the coexistence of these two pheasants. Furthermore, we found that habitat partitioning associated with ridge crests facilitates the coexistence of these species. These findings support habitat partitioning hypotheses.

Elevation was the most important environmental determinant of habitat suitability for both species at both the meso‐scale and macro‐scale. This result concurs with the strong preference of these two species for high‐elevation habitats. Both *L*. *lerwa* and *T*. *tibetanus* are typical high‐elevation alpine birds (Li & Lu, 1992) with some seasonal movement along the altitudinal gradient (Cheng et al., 1978; Zheng, 2016). The species move to lower elevations near the tree line in winter and to higher elevations on alpine shrub and grasslands or alpine meadows near the snow line in the summer breeding period because food is more abundant (Yao et al., 2017; Zheng, 2016). Such confined niches lead these species to select high‐elevation habitats. However, we found that *T*. *tibetanus* occupies a wider elevational range than *L*. *lerwa* (Table 1), particularly at high elevations. *Tetraogallus tibetanus* was found at elevations ranging from 5500 m to 6000 m (Cheng et al., 1978; Zheng, 2016). According to our field observations, the geographic ranges of these two species are closely related to the snow line. *Tetraogallus tibetanus* extends to a higher elevation than *L*. *lerwa* due to the elevation of the snow line because the snow line in the hinterland of the plateau is higher than that at the southeastern margin of the QTP (Wu, 1989). Wu (1989) also showed that in the hinterland of the plateau, the modern snow line was at about 5200 m, while in the Bomi‐Chayu‐Yulong bend of the southeastern plateau, the snow line is lower at 4300 m. Furthermore, the snow line has been highlighted as an important climate boundary, which is sensitive to changes in temperature, precipitation, and topography (Fang et al., 2011).

Our study indicated that factors related to topography, temperature variation, and precipitation contributed to the coexistence of *L*. *lerwa* and *T*. *tibetanus* under the high‐elevation conditions on the QTP. At the meso‐scale, our study showed that *T*. *tibetanus* habitats overlapped with those of *L*. *lerwa*, particularly in Xiaojin. This result agrees with that of a previous investigation in the Longmen mountains, which concluded that *L*. *lerwa* and *T*. *tibetanus* lived in the same habitats (Li & Lu, 1992). Elevation, vegetation, aspect, and slope influenced the suitable habitats of both *L*. *lerwa* and *T*. *tibetanus*. As omnivorous pheasants, these species feed on plants, mainly underground stems, roots, and tubers, and some small invertebrates (Cheng et al., 1978; Yao et al., 2017). Alpine meadows provide suitable food sources, and sunny slopes support more abundant plants compared to shady slopes, which aids foraging (Pu et al., 2011; Yao et al., 2017). Thus, similarities in the characteristics that are important for habitat suitability between these two species lead to the similar habitat uses. However, stable coexistence requires habitat (i.e., niche) or resource differences between species (Chesson, 2000). We found differences in the habitats selected by these species in terms of topographic factors related to ridge crests, which were not influential for *T*. *tibetanus* but were the most influential for *L*. *lerwa*. We found that *L*. *lerwa* were most active on steep terrain directly on the ridges, which provides a greater field of view and easier ground on which to take off for gliding and escaping from predators (Yao et al., 2017). Conversely, *T*. *tibetanus* was found to range further away from such ridge crests. *Tetraogallus tibetanus* are larger than *L*. *lerwa*, and this larger body size could lead to *T*. *tibetanus* moving within greater ranges. A previous study suggested that body size differences among sympatric bird species facilitate community coexistence (Leyequién et al., 2007). Therefore, we consider that the differences in the use of ridge crests help *T*. *tibetanus* and *L*. *lerwa* to coexist in sympatry. Habitat partitioning based on ridge crests serves to reduce both interference and competition, facilitating the coexistence of ecologically similar species. Habitat partitioning is a widespread form of niche divergence adopted by sympatric mammals and birds that allows the coexistence of related species (Byrne et al., 2019; Cui et al., 2008; Namgail et al., 2004; Wei et al., 1999). An et al. (2009) suggested that the current geographic range of *T*. *tibetanus* has been influenced by the most recent large‐scale glaciations on the QTP in and before the Pleistocene. Population expansion occurred from the Qilian Mountains where the population of *T*. *tibetanus* shows high genetic diversity (An et al., 2009). The Qilian Mountains are in the northeast of the QTP toward the core of the Chinese mainland, where the temperature varies widely in a typical continental climatic regime (Wei et al., 2017). According to Cai et al. (2018), *Tetraogallus* is one of a cluster of related genera that might have arisen in Africa or broadly across the Eurasian and African land masses. Five allopatric members of the genus now occur in the Palaearctic from the Caucasus and Caspian to Mongolia and Siberia (Vaurie, 1959). The bulk of their geographic range is thus more northerly and westerly, whereas *L*. *lerwa* is a Sino–Himalayan species located along the south of the QTP (Cai et al., 2018; Vaurie, 1972). Hence, past geographic range changes affected by geographical origin and climatic fluctuations have resulted in areas of overlap along the southeastern margin of the QTP.

At the macro‐scale, temperature variation and precipitation mainly influenced the habitat suitability of *L*. *lerwa* and *T*. *tibetanus*. Researchers have found that *T*. *tibetanus* adapted to cold and harsh alpine conditions (An et al., 2009). *Tetraogallus tibetanus* is distributed across a wider range that not only covers most of the southeastern margin of the QTP inhabited by *L*. *lerwa* but also extends into the drier and colder hinterland of the plateau, which could be consistent with the species’ adaption to drier and colder conditions. The southeastern margin of the QTP is rainy and humid because of the influence of monsoons from the Indian Ocean (Hamerlík & Jacobsen, 2012; Xu et al., 2009). Furthermore, the occurrence and development of hot conditions and low atmospheric pressure in the summer on the QTP are strengthened by the wet southwest monsoon season (Wu, 1989). During this season (mainly July to August), the southeastern region is humid or semi‐humid due to high levels of precipitation near the boundary (Wu, 1989). Research has also shown that the mean annual temperature in the southeast of plateau is 7–9°C higher than that in the eastern regions at the same latitude and height and that annual differences in temperature within the southeastern plateau were less variable (Wu, 1989). Therefore, *L*. *lerwa* could adapt to warm and humid alpine conditions, which led to this species being more influenced by precipitation than *T*. *tibetanus*. Consequently, we found a lower abundance of *T*. *tibetanus* than *L*. *lerwa* in our repeated field surveys in the Balangshan mountains, which was similar to previous field observations in the Longmen mountains (Li & Lu, 1992). This is because both the Balangshan and Longmen mountains are located along the southeastern edge of the QTP with warm and rainy conditions, where habitats are more suitable for *L*. *lerwa* than *T*. *tibetanus*.

Differences in nesting behaviors and reproductive season between species in sympatric ranges could facilitate their coexistence by maximizing fitness (Li & Wang, 2021). Recent research reported hatching data for *L*. *lerwa* from June 21 to July 10 (Li et al., 2022) while *T*. *tibetanus* have finished hatching in early June (Li & Wang, 2021) in Basu County and Cuona County in the Tibet Autonomous Region, China (southeastern QTP). The egg laying period of *T*. *tibetanus* has been reported in mid‐May in Tibet (Zheng, 2016). Li and Wang (2021) also found that *L*. *lerwa* nests are cave nests in alpine shrubs, grasslands, or alpine meadows, while *T*. *tibetanus* nests are ground nests in bare rock areas or alpine shrubs. This difference in nesting behavior could reduce competition for resources and facilitate coexistence. Therefore, we inferred these differences in nest type, habitat use, and reproductive season between *L*. *lerwa* and *T*. *tibetanus* could be an adaption strategy for species coexistence under high‐elevation conditions.

We found that the difference in spatial ranges between *L*. *lerwa* and *T*. *tibetanus* on the QTP was not caused by climatic niche differentiation. The environmental niches of the two species were more similar than expected under the null hypothesis, but they were rarely identical (Warren et al., 2008). Thus, to some extent, conserved niches between two species allow differences in habitat suitability. Therefore, we could conclude that coexistence of *L*. *lerwa* and *T*. *tibetanus* at high elevations is related to specific habitat or resource differences in terms of topographical factors (i.e., elevation and ridge crests) and climatic conditions (i.e., temperature and precipitation), as well population expansion history and different breeding strategies.

*Lerwa lerwa* and *T*. *tibetanus* are the only two Galliformes in this high‐elevation habitat and appear to represent the majority of the standing avian biomass throughout the year. Other bird species present on the QTP are typically smaller, occur in smaller numbers, or are on passage (e.g., accentors and rosefinches). Larger birds (e.g., vultures and eagles) are scarce, intermittently present, and have large movement ranges, so their standing biomass is low. *Lerwa lerwa* and *T*. *tibetanus* are predominately dependent on vegetation for food, which is limited with low species diversity and biomass and simplified vegetation structure. There are some limitations to the present study. For example, our results do not reflect the Eltonian niche, which includes diet and predators (Elton, 1927). Eltonian niches are difficult to measure at broad geographic scales due to the dynamic and complex axes of multidimensional change (Soberón, 2007). Although the ecological niches of these two species were thought to be quite similar in Balangshan and Longmen mountains (Li & Lu, 1992), it is possible that their food niches have differentiated or they show clearly different diets or foraging behaviors (e.g., one feeding on foliage and the other feeding on tubers or roots). However, this does not appear to be the case based on our observations. To further understand the link between habitat suitability and ecological niche, the diet and food requirements of *L*. *lerwa* and *T*. *tibetanus* should be considered in future studies. In addition, global climatic warming in the southeastern QTP has been much greater than that in other mountainous ecosystems, and high‐elevation systems are at greater risk (Islamia & Delhi, 2013). Therefore, *L*. *lerwa* and *T*. *tibetanus* should be focused on in future studies as they are at higher risk of suitable habitat changes caused by climate change and possible mortality, particularly *L*. *lerwa*, which is more closely related to the snow line.

In conclusion, our findings confirmed that habitat partitioning related to topography (i.e., ridge crests) facilitates the coexistence of the only two Galliformes, *L*. *lerwa* and *T*. *tibetanus*, in sympatric ranges on the QTP. Temperature, precipitation, and elevation were the major factors accounting for the habitat suitability differences between these two species under the high‐elevation conditions. *Tetraogallus tibetanus* extended into the hinterland of the plateau and occurred at higher elevations, which encompassed a broader temperature range along with colder and drier alpine conditions with less glacial coverage and less monsoonal influence. Conversely, *L*. *lerwa* occurs along the southeastern margin of the QTP with a lower snow line, an area prone to rainy and humid conditions. That these two pheasants coexist in sympatry in high‐elevation conditions could be an adaption to differences in alpine conditions.

## CONFLICT OF INTEREST

There are no conflicts of interest to declare.

## AUTHOR CONTRIBUTIONS

**Hongyan Yao:** Conceptualization (equal); Data curation (lead); Formal analysis (lead); Investigation (lead); Methodology (lead); Resources (equal); Software (equal); Validation (lead); Writing – original draft (lead); Writing – review & editing (lead). **Pengcheng Wang:** Data curation (supporting); Formal analysis (supporting); Methodology (supporting); Software (supporting); Writing – original draft (equal); Writing – review & editing (equal). **Geoffrey Davison:** Data curation (equal); Investigation (equal); Writing – original draft (equal); Writing – review & editing (equal). **Yong Wang:** Writing – original draft (supporting); Writing – review & editing (equal). **Philip J. K. McGowan:** Writing – original draft (supporting); Writing – review & editing (equal). **Nan Wang:** Conceptualization (equal); Funding acquisition (lead); Investigation (equal); Resources (equal); Supervision (equal); Validation (equal); Writing – original draft (equal); Writing – review & editing (equal). **Jiliang Xu:** Conceptualization (equal); Project administration (equal); Supervision (equal); Writing – original draft (supporting); Writing – review & editing (equal).

## Supporting information

Figure S1

Figure S2

Figure S3

Figure S4

Figure S5

Appendix S1

Appendix S2

Appendix S3

## Data Availability

Occurrence data of two species at both two scales are available at the Dryad (https://doi.org/10.5061/dryad.vt4b8gtt6).
