# Supplementary figures and images for: How do Snow Partridge (Lerwa lerwa) and Tibetan Snowcock (Tetraogallus tibetanus) coexist in sympatry under high‐elevation conditions on the Qinghai–Tibetan Plateau?
Source: Ecol Evol. 2021 Dec 8;11(24):18331–41. doi: 10.1002/ece3.8424 (PMC8717327; doi:10.1002/ece3.8424)

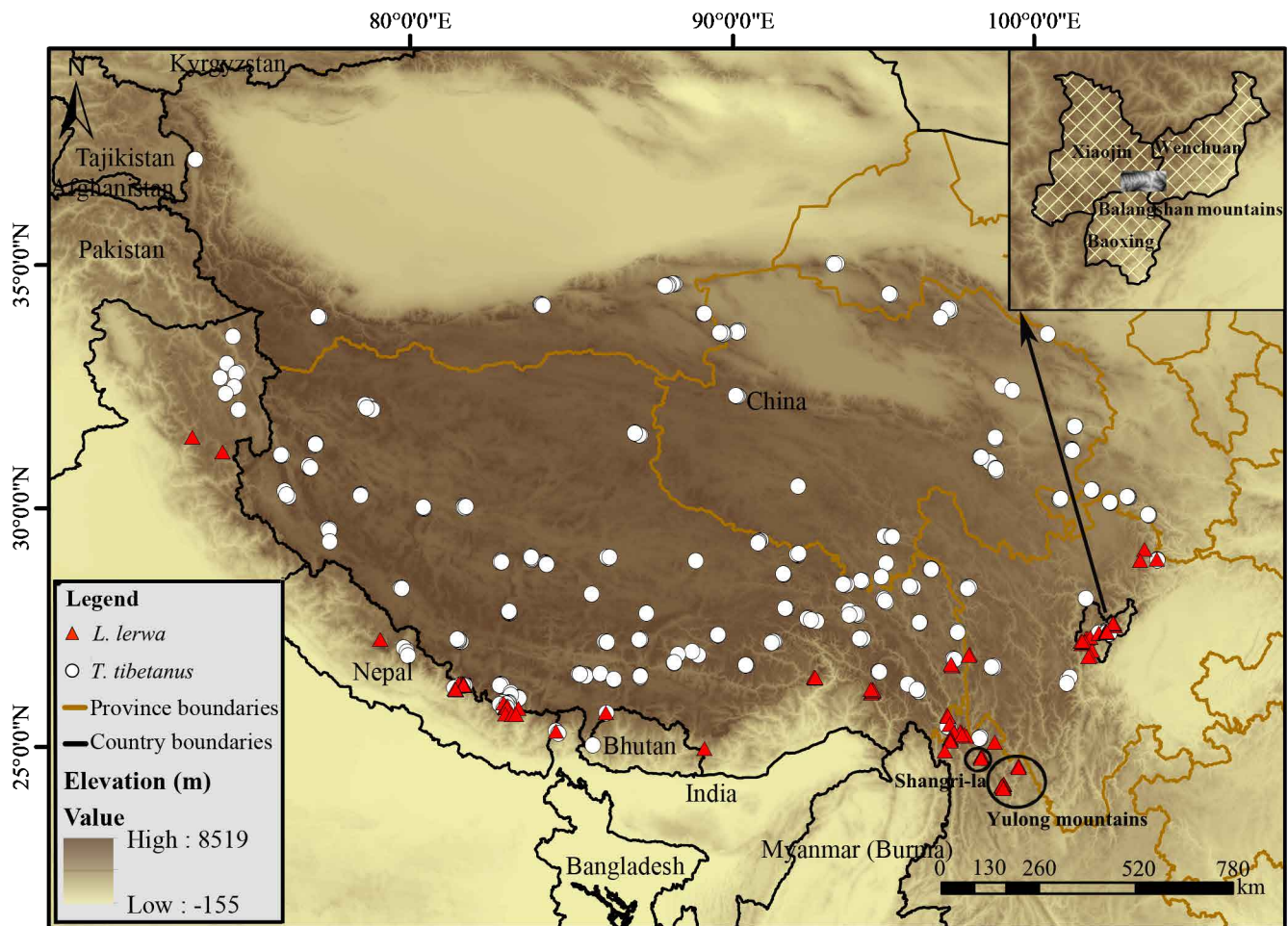

Supplement: Supplementary file 1 — Figure S1 [file ECE3-11-18331-s004.pdf]

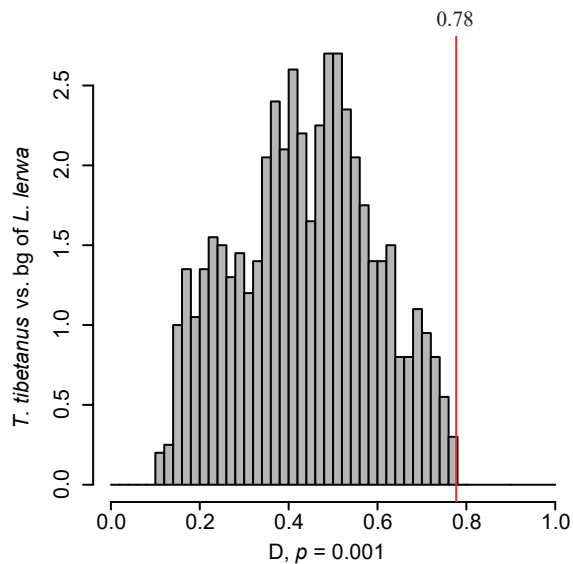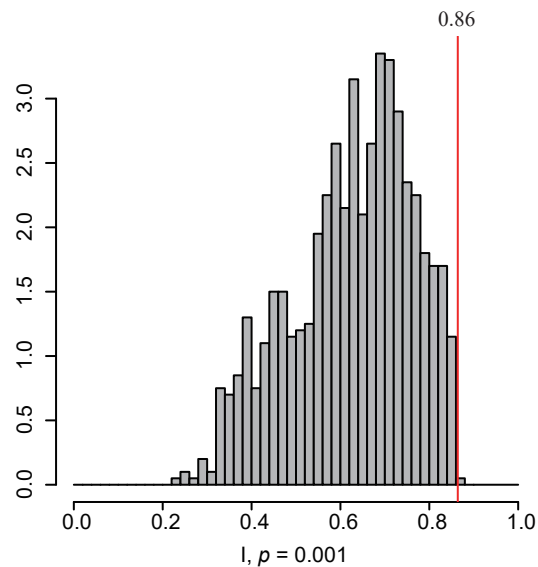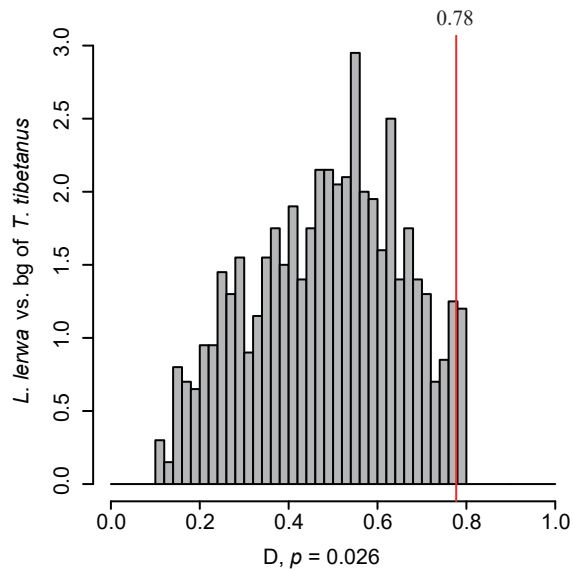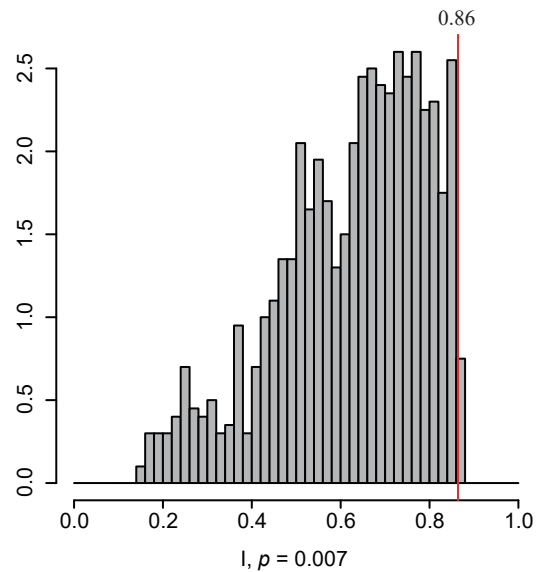

Supplement: Supplementary file 2 — Figure S2 [file ECE3-11-18331-s007.pdf]

*T. tibetanus* vs. bg of *L. lerwa*

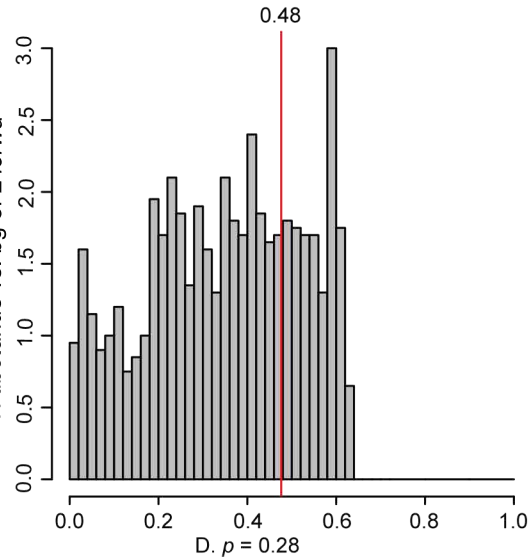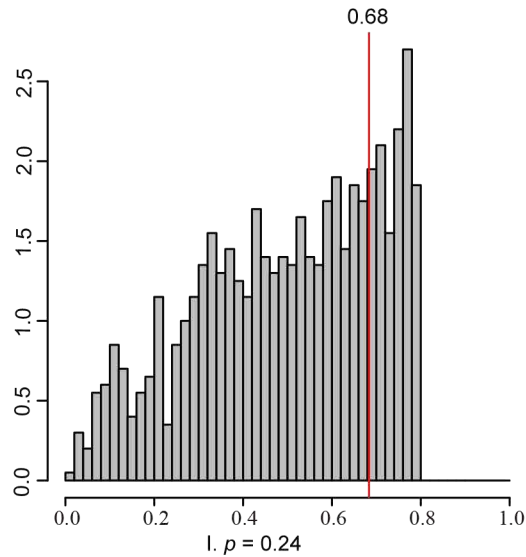

*L. lerwa* vs. bg of *T. tibetanus*

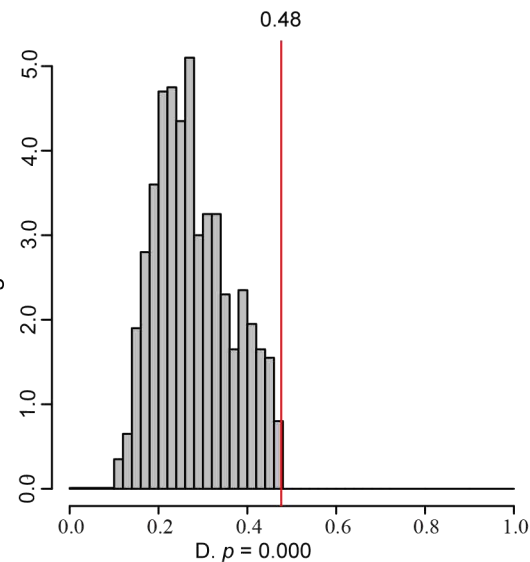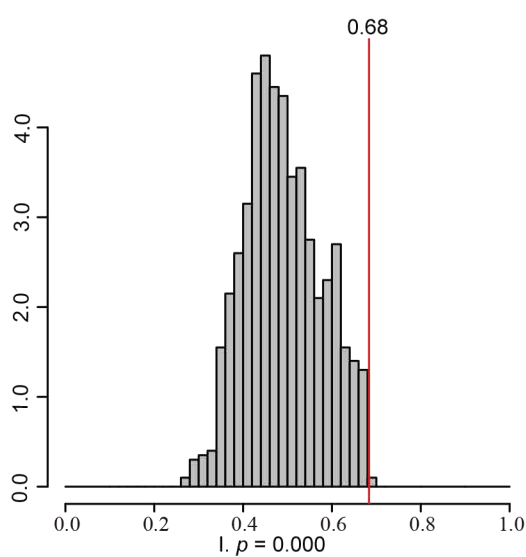

Supplement: Supplementary file 3 — Figure S3 [file ECE3-11-18331-s006.pdf]

Density

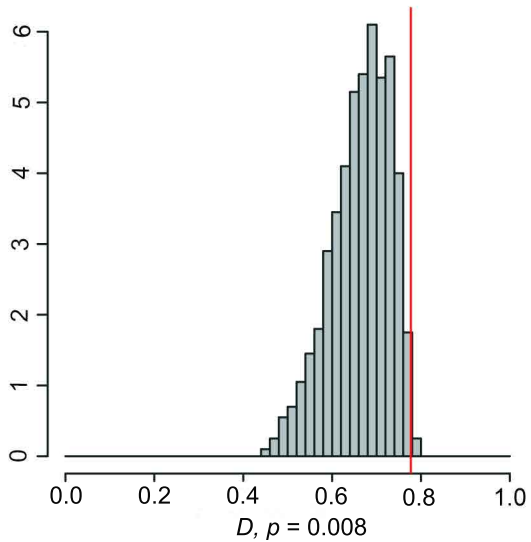

Density

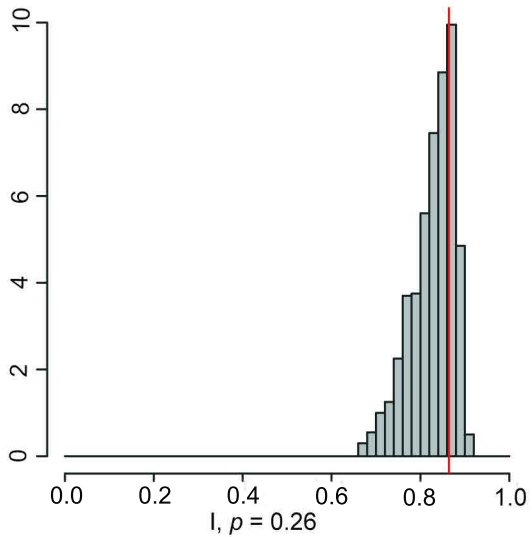

Supplement: Supplementary file 4 — Figure S4 [file ECE3-11-18331-s008.pdf]

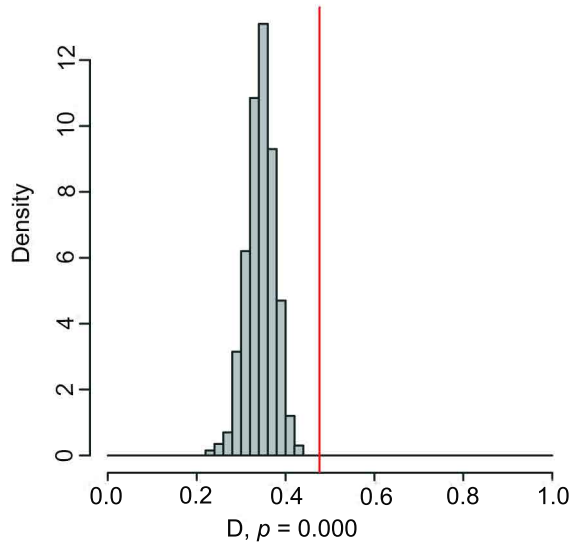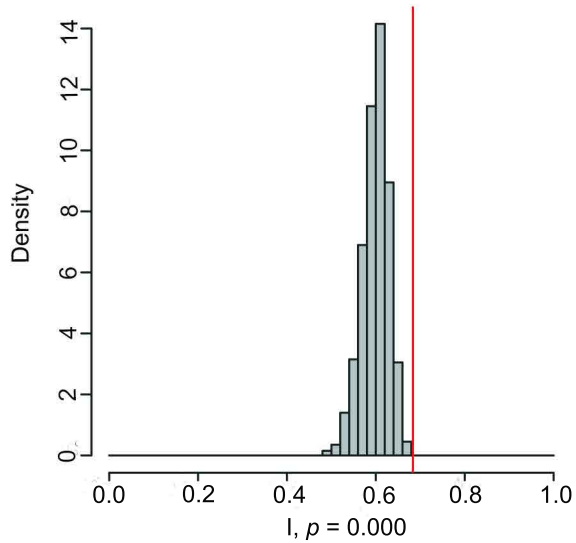

Supplement: Supplementary file 5 — Figure S5 [file ECE3-11-18331-s002.pdf]
