# Supplementary material for: How do Snow Partridge (Lerwa lerwa) and Tibetan Snowcock (Tetraogallus tibetanus) coexist in sympatry under high‐elevation conditions on the Qinghai–Tibetan Plateau?
Source: Ecol Evol. 2021 Dec 8;11(24):18331–41. doi: 10.1002/ece3.8424 (PMC8717327; doi:10.1002/ece3.8424)
Supplement: Supplementary file 6 — Appendix S1 [file ECE3-11-18331-s005.docx]

**Appendix S1**

**Methods**

**Environmental variables for ENMs at the meso-scale**

At meso-scale, we chose environmental variables potentially important for *T. tibetanus* including elevation, slope and aspect, distance to the nearest ridge crest, and vegetation (Cheng et al. 1978), which were also used for habitat suitability prediction of *L. lerwa* in the three counties area (total land area 12,800 km^2^) (Yao et al., 2017) because *T. tibetanus* and *L. lerwa* used the same habitats where they are sympatric. These variables were ranked three groups. The first group, referred to as terrain, consisted of elevation, slope (degrees from horizontal) and aspect (compass degrees) from the digital elevation model (DEM) which was from the Geospatial Data Cloud of the Chinese Academy of Sciences (http://www.gscloud.cn/). The variable aspect, reclassified to eight direction codes as North 1, Northeast 2, East 3, Southeast 4, South 5, Southwest 6, West 7 and Northwest 8, in 45° segments from 0° to 360° clockwise, with 0° and 360° both indicting due the north, is a categorical environmental layer in modeling. The second group of variables, referred to and based on distances, consisted of the distance to the nearest ridge, digitized and transformed from ridge lines by the Spatial Analyst Tools of ‘Euclidean Distance’. The ridge lines were extracted from a hydrological analysis by the DEM of the Geospatial Data Cloud of the Chinese Academy of Sciences in ArcGIS 10.2. The third group was the vegetation type derived from the National Geomatics Center of China (<http://www.globallandcover.com/>).

All variables were at a resolution of 30 m × 30 m, within the Projected Coordinate Systems of the Beijing 1954 GK Zone 18 N from the Geographic Coordinate Systems of the WGS 1984. We used the Band Collection Statistics in the Spatial Analyst extension ArcGIS to calculate correlation among the five environmental layers (Lu et al., 2012); no variable was eliminated because the highest correlation was only 0.41 between ‘ridgedis’ and ‘vegetation’ (Table S1 in Appendix S3).

**VIF analysis for multicollinearity and Person’s correlation for collinearity between variables at the macro-scale**

Among the 20 predictors, strong multicollinearity could result in overfitting the ENMs. We have considered using Variance Inflation Factor (VIF) to detect multicollinearity. After VIF analysis, eight variables were remained with correlation coefficients < |0.80|: mean diurnal range (mean of monthly (max temp - min temp), MDR, ℃), Isothermality (IS), mean temperature of wettest quarter (MTWQ, ℃), mean temperature of driest quarter (MTDQ, ℃), precipitation in driest month (PDR , mm), precipitation seasonality (PS, coefficient of variation), precipitation of warmest quarter (PWQ, mm) and precipitation of coldest quarter (PCQ, mm). This study aims to explore how two pheasants coexist under high-elevation conditions in the QTP. Elevation as a potential important factor would be better remained. Besides, species richness usually is determined by mulit-factors such as climate-energy and habitat heterogeneity (Jimenez-Alfaro et al., 2016; Moura et al., 2016). We used Pearson’s correlations to detect collinearity between every two predictors, and finally also eight variables were remained with a Pearson’s correlation < |0.80|: elevation (Ele, m), mean diurnal range (mean of monthly (max temp − min temp), MDR, ℃), temperature seasonality (TS, standard deviation *100), mean temperature of driest quarter (MTDQ, ℃), annual precipitation (AP, mm), precipitation in driest month (PDM, mm), precipitation seasonality (PS, coefficient of variation), and precipitation in coldest quarter (PCQ, mm). Among these eight variables, five are consistent with remained factors by VIF: MDR, MTDQ, PDM, PS, and PCQ. The eight variables selected based on Pearson’s correlation are related to elevation, temperature variation and precipitation variation which could reflect habitat heterogeneity and climate–energy. These eight variables are more important in biological and ecological meaning for these two pheasants and could meet our aim, though the remained variables by VIF would be important in statistics. Therefore, we finally used Pearson’s correlations to detect collinearity and deleted variables.

**Species data of *L. lerwa* and *T. tibetanus* at the whole-of distribution scale**

At the macro-scale of the QTP, species occurrences were recorded from field survey, published references and online databases of the Global Biodiversity Information Facility (GBIF, <http://www.gbif.org/>). For *L. lerwa*, 104 occurrences were used for habitat suitability prediction, of which 10 points were from field survey records, 84 were from previous references (Cheng et al., 1965; Li, 1986; Lu et al., 1988；Lu et al., 1989；Han et al., 2004；Zhou et al., 2004a; Li et al., 2010; Liu et al., 2010；Srivastava & Dutta, 2010；Jia, 2012；Khanal et al., 2012; Li et al., 2013; Luo, 2013; Yang & Wang, 2013; Xiao et al., 2014; Fig. 1), and 10 were from GBIF. For *T. tibetanus*, 328 occurrences were used for habitat suitability prediction, of which 16 points were from field survey records, 166 from previous references (Lee et al., 1965; Cheng et al., 1965; Zheng & Pi, 1979; Yang & Xu, 1987; Pu, 1990; Wei, 1990; Ma et al., 1991; Yu et al., 1993; Ma, 1997; Tashi et al., 2004; Zhou et., 2004a; Zhou et., 2004b; Pu et al., 2011; Fig. 1), and 146 were from GBIF.

**References**

Cheng, T. S., Tan, Y. K., Lu, T. C., Tang, C. Z., Bao, G. J., & Li, F. L. (1978). Fauna a, Aves: Galliformes (Vol. 4, pp. 48–51). Beijing: Science Press.

Cheng, Z. X., Tan., K. Y., & Li., Y. X. (1965). Surveys on avifauna on northwesten Sichuan. *Acta Zoological Sinica*, 4: 435-448.

Han, Z. X., Yang, J. D., & Hu, J. C. (2004). Preliminary report on rare animal resources in Fengtongzhai Nature Reserve. *Journal of Gansu Lianhe University (Natural Sciences)*, 18, 58-60.

Jia, D. F. (2012). Research on the Priority Protection Area of Rare, Endangered, and Endemic Birdson the Qinghai-Tibet Plateau. Beijing Forestry University.

Jimenez-Alfaro, B., Chytry, M., Mucina, L., Grace, J. B. & Rejmanek, M. (2016). Disentangling vegetation diversity from climate–energy and habitat heterogeneity for explaining animal geographic patterns. *Ecology and Evolution*, 6, 1515-1526.

Khanal, B., Chalise, M. K., & Solanki, G. S. (2012). Diversity of butterflies with respect to altitudinal rise at various pockets of the Langtang National Park, central Nepal. *International Multidisciplinary Research Journal*, 2, 41-48. <https://updatepublishing.com/journal/index.php/imrj/article/view/1557>.

Lee, T. H., Cheng, S. W., & Cheng, T. H. (1965). Avifaunal studies of the Yuh-shuh autonomous region, Qinghai Province. *Acta Zoologica Sinica,* 17, 217-229.

Li, J. J., Han, X. L., Cao, H. F., Tian, Y., Peng, B. Y., Wang, B., & Hu, H. J. (2013). The fauna and vertical distribution of birds in Mount Qomolangma National Nature Reserve. *Zoological Research*, 34, 531-548. <https://doi.org/10.11813/j.issn.0254-5853.2013.6.0531>

Li, S., McShea, W. J., Wang, D J.., Shao, L. K., & Shi, X. G. (2010) The use of infrared-triggered cameras for surveying phasianids in Sichuan Province, China. *Ibis*, 152, 299–309. <https://doi.org/10.1111/j.1474-919X.2009.00989.x>

Li, X. T. (1986). Sidelight of breeding information on Beichuan County’s rare pheasants. *Sichuan Journal of Zoology*, 38–9.

Liu, L. M., Dong, F., He, R. H., Wu, F., Wang, K., Kong, D. J., …Yang, X. J. (2010). Bird Resources of Yulong Snow Mountain Nature Reserve in Lijiang of Yunnan. *Sichuan Journal of Zoology*, 29, 232-239.

Lu, N., Jing, Y., Lloyd, H. & Sun, Y. H. (2012). Assessing the distributions and potential risks from climate change for the Sichuan Jay (*Perisoreus internigrans*). *The Condor*, 114, 365–376.

Lu, T. C. (1988). Surveys on the ecology and the vertical distribution of pheasants in Baoxing County, Sichuan Province. *Zoological Research*, 9, 37–44.

Lu, T. C., Liu, R. S., & He, F. Q. (1989). Survey on pheasants in Aba, Ganzhou and Liangshan of Sichuan. *Journal of China West Normal University (Nature Science)*, 2, 7–14.

Luo, H. B. (2013). Biodiversity and Protective Countermeasures for Ranwu Lake Wet Land Nature Reserve of Tibet. *Central South Forest Inventory and Planning*, 38-41.

Ma, M., Zhou, Y. H., & Ma, L. (1991). Distribution and Ecological observation of Tibetan Snowcock in Xinjiang. *Chinese Journal of Wildlife*, 4, 15-16.

Ma, S. (1997). The Breeding Active Regularity of Tibetan Snowcock. *Acta Agriculturae Boreali-occidentalis Sinica*, 6, 8-10.

Moura, M. R., Villalobos, F., Costa, G. C. & Garcia, P. A. (2016). Disentangling the role of climate, topography and vegetation in species richness gradients. *Plos One*, 11(3): e0152468.

Pu, B., Zhaxi, L. J., La, D., & Ba, S. (2011). Study on Behavior and Feeding Site Selection of Tibetan Snowcock (*Tetraogallus tibetanus*) during the Winter. *Journal of Tibet University (Natural Sciences)*, 26, 1-6. <https://doi:10.16249/j.cnki.54-1034/c.2011.02.033>

Pu, R. Z. (1990). Estimation of density and population size for Tibetan Snowcock. *Acta Zoologica Sinica*, 36, 433-435.

Srivastava, T., & Dutta, P. K. (2010). Snow Partridge hopes for a safe home in Arunachal Pradesh. *Current Science*, 98, 289.

Tashi, T. L., Lha, Q., Duan, S. Q., & Liu, S. C. (2004). Preliminary Report on Captive Raising and Breeding Tibetan Snowcock. *Chinese Journal of Zoology*, 39, 48-51. <https://doi.org/10.13859/j.cjz.2004.01.010>

Wei, J. G. (1990). Breeding habits and artificial domestication of Tibetan Snowcock. *Chinese Journal of Wildlife*, 2, 31-33.

Xiao, Z. S., Hu, L., Wang, X., Shang, T., Zhu, D. H., Zhao, Z. L., & Huang, X. Q. (2014). Wildlife diversity after the Wenchuan Earthquake: a case from the Guangguangshan Valley of Longchihongkou National Nature Reserve, Southwest China. *Biodiversity Science*, 22, 1–4. https://doi: 10.3724/SP.J.1003.2014.14209

Yang, L., & Xu, Y. G. (1987). A new subspecies of the Tibetan Snowcock – *Tetraogallus tibetanus yunnaneusis* (Galliformes: phasianidae). *Acta Zootaxonomica Sinica*, 1, 024.

Yang, P. F., & Wang, D. (2013). Research and Investigation on the Vegetation Type of Quzong Gong Golden Girl River Basin in Northern White Horse Snow Mountain Nature Reserve. *Forest Inventory and Planning*, 38, 101-106. <https://doi.org/10.3969/j.issn.1671-3168.2013.04.020>

Yao, H. Y., Davison, G., Wang, N., Ding, C. Q., & Wang, Y. (2017). Post‑breeding habitat association and occurrence of the Snow Partridge (*Lerwa lerwa*) on the Qinghai‑Tibetan Plateau, west central China. *Avian Research*, 8, 8. <https://doi.org/10.1186/s40657-017-0066-5>

Yu, S. F., Ma, S., Ren, Z. H., Xu, S. C., Yang, M. S., & Yuan, N. (1993). Observation of Breeding Activities of Tibetan Snowcock. *Journal of Qinghai Animal Husbandry and Veterinary Medicine College*, 10, 1-4.

Zheng, S. W., & Pi, N. L. (1979). Preliminary observation on the ecology of Tibetan Snowcock. *Chinese Journal of Zoology*, 1, 29. <https://doi.org/10.13859/j.cjz.1979.01.010>

Zhou, Y., Zhang, J., Zhang, W., Zhou, C., Hu, J., & Zhang, J. (2004a). Resources and Preserve of Birds in Xiaozhaizigou Nature Reserve. *Journal of China West Normal University (Natural Sciences)*, 25, 68-72. <https://doi.org/10.16246/j.issn.1673-5072.2004.01.017>

Zhou, J., Guo, Y., Mi, Z., Hu, J., & Zhou, C. (2004b). Preliminary Study on Digestive System of Tetraogallus tibetanus in Morphology. *Journal of China West Normal University (Natural Sciences)*, 25, 415-417. <https://doi.org/10.16246/j.issn.1673-5072.2004.04.019>
