# Supplementary material for: How do Snow Partridge (Lerwa lerwa) and Tibetan Snowcock (Tetraogallus tibetanus) coexist in sympatry under high‐elevation conditions on the Qinghai–Tibetan Plateau?
Source: Ecol Evol. 2021 Dec 8;11(24):18331–41. doi: 10.1002/ece3.8424 (PMC8717327; doi:10.1002/ece3.8424)
Supplement: Supplementary file 7 — Appendix S2 [file ECE3-11-18331-s001.docx]

**Appendix S2:**


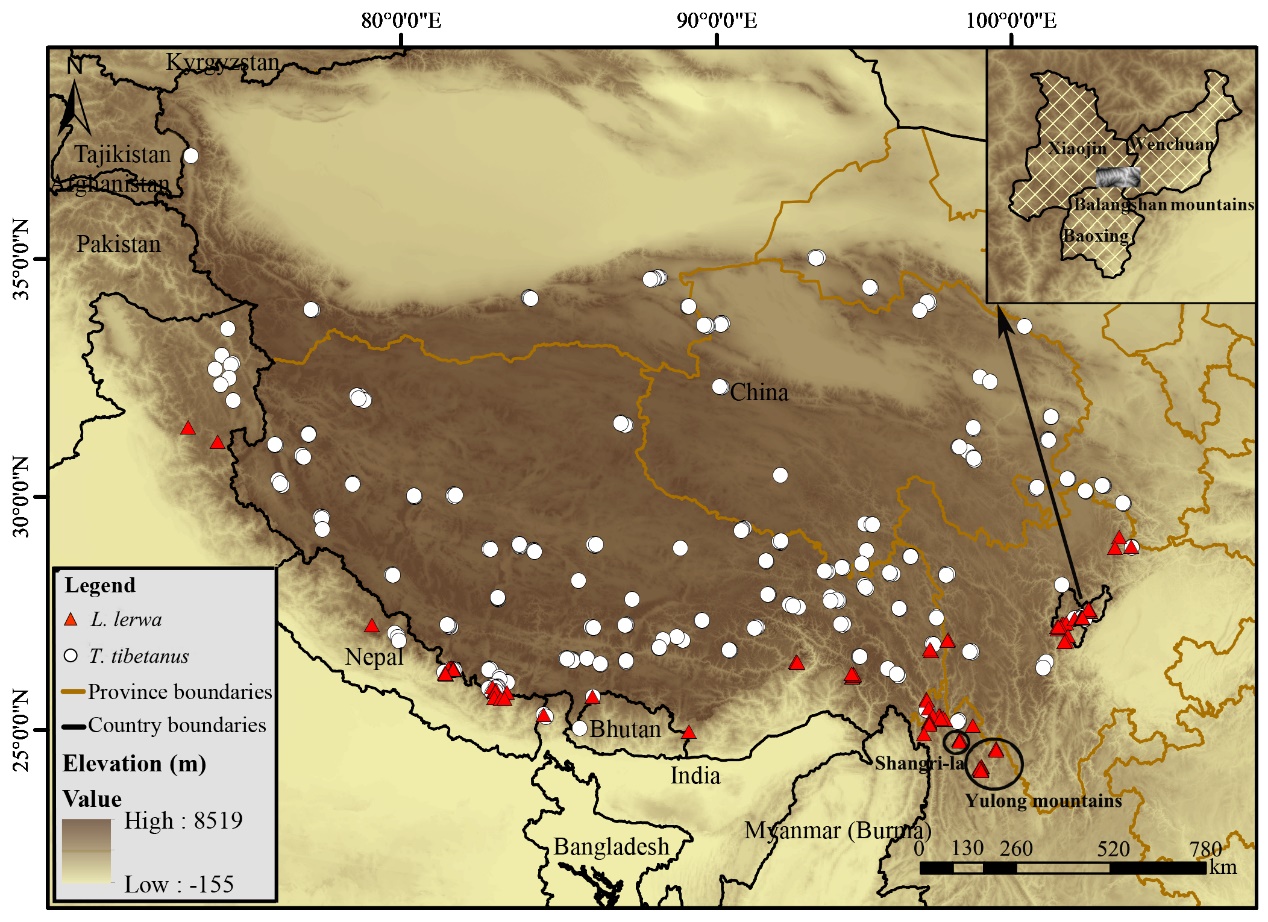


Figure S1. The 104 occurrences of *Lerwa lerwa* and 328 occurrences of *Tetraogallus tibetanus* were used for habitat suitability predictions, respectively. Black circles signed locations of two mountains of Shangri-la (left small circle) and Yulong mountains (right big circle). Black box pointed out by arrow indicates the meso-scale area within the three counties (Baoxing, Xiaojin and Wenchuan), Sichuan Province, China, within which the central black range shows the Balangshan mountains where we conducted field survey in August 2013.


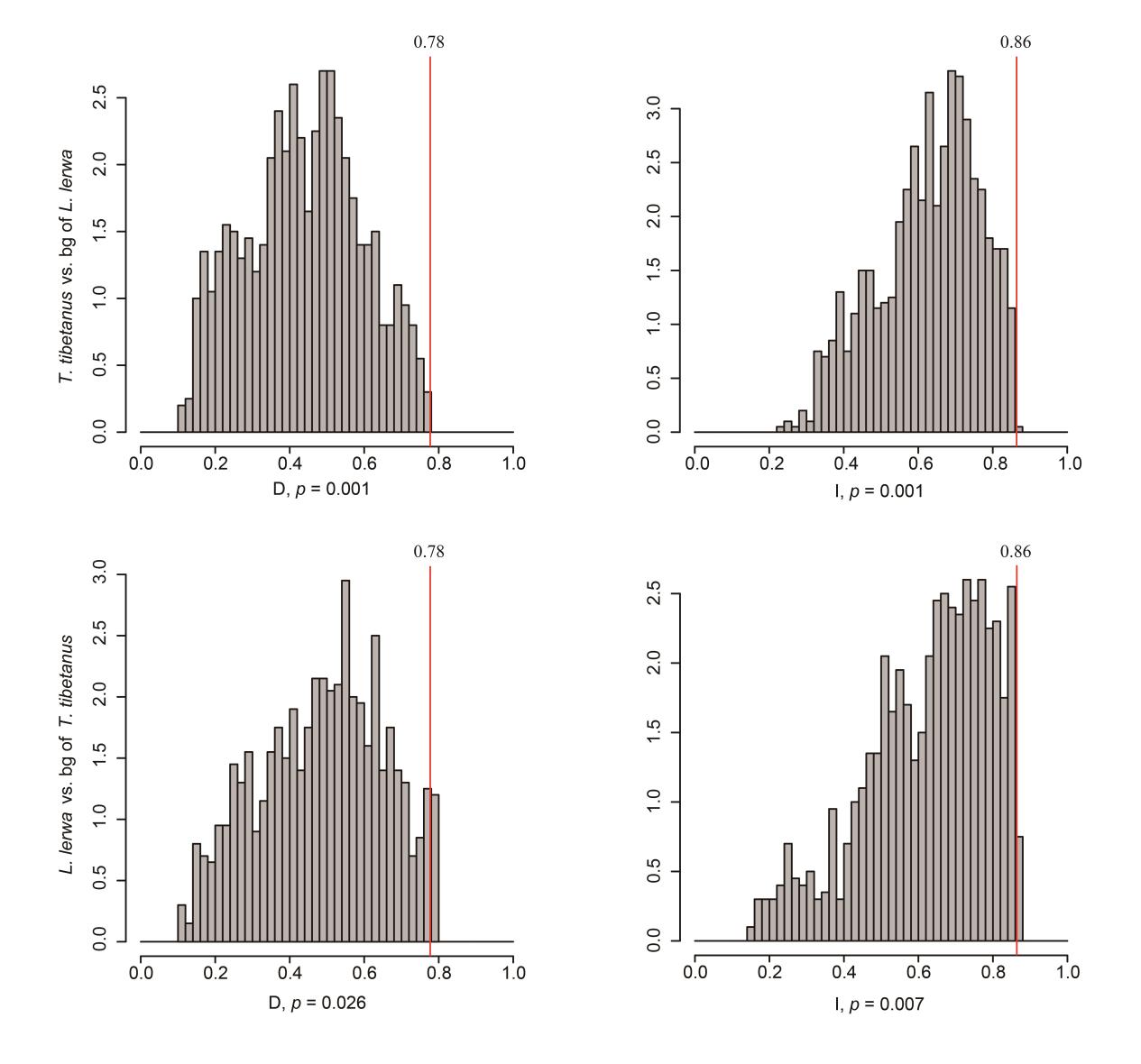


Figure S2. The results of the niche similarity test between *Lerwa lerwa* and *Tetraogallus tibetanus* at meso-scale. If the value of niche similarity indices *D* and *I* are close to 1, the niche is similar; if their value is near 0, the niche is different. The two niche similarity indices are both on the right side of 95 % confidence intervals (columnar in grey) suggesting that the niches are significantly similarity (*p < .05*). By contrast, the *D* and *I* values are on the right side of 95 % confidence intervals, indicating that the niches are significantly conserved (*p < .05*).


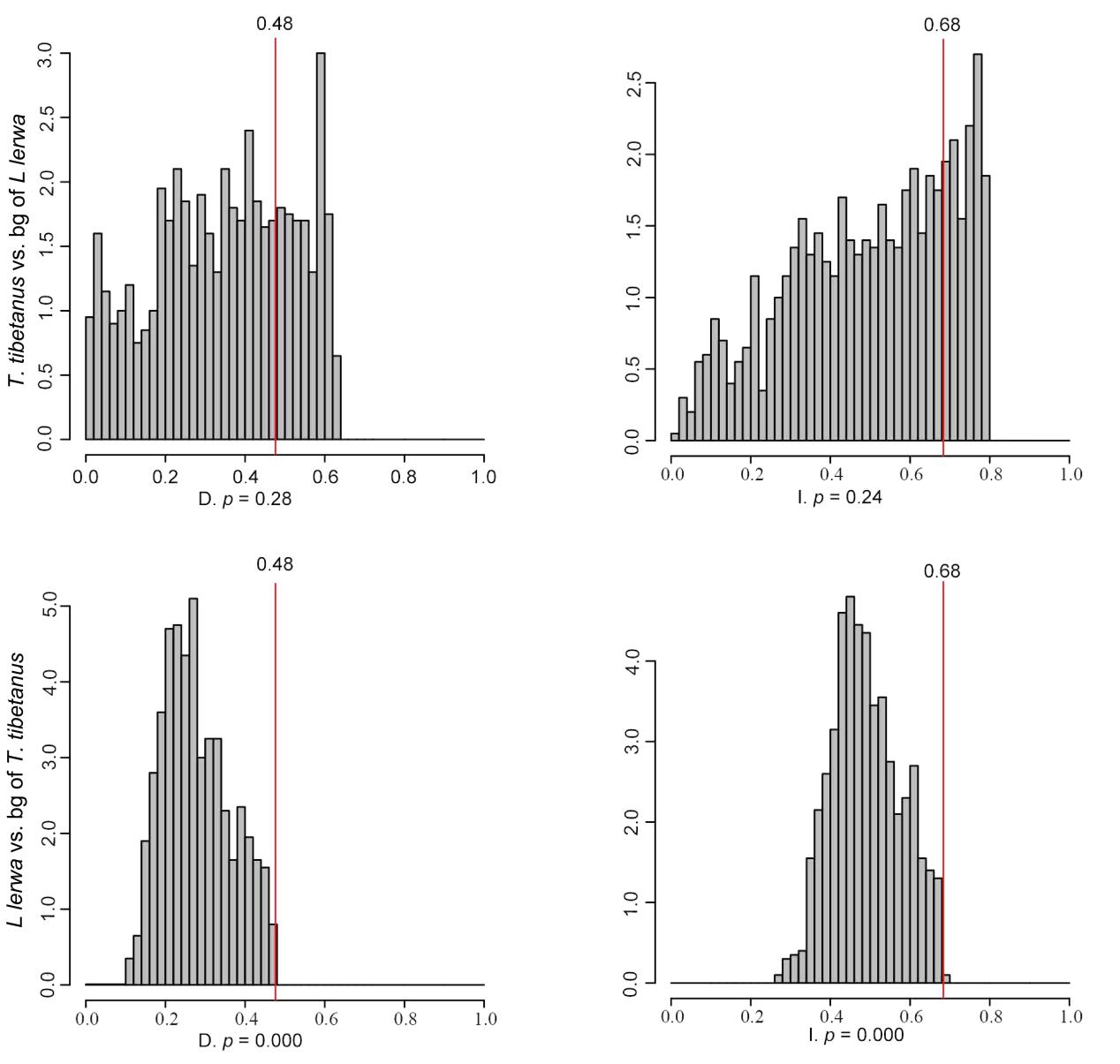


Figure S3. The results of the niche similarity test between *Lerwa lerwa* and *Tetraogallus tibetanus* at the whole-of-distribution scale. The ecological niches were significantly similar when range of *T. tibetanus* as for background test with *D* and *I* to the right side of the 95 % confidence interval (*p < .05*).


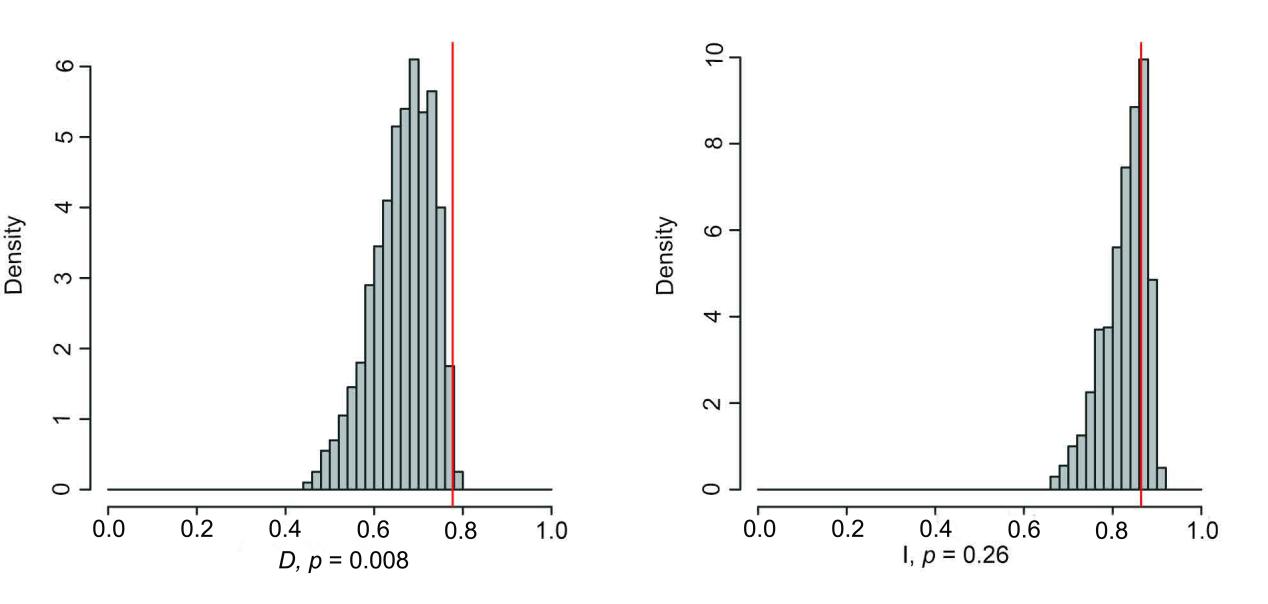


Figure S4. The results of the niche equvalency test between *Lerwa lerwa* and *Tetraogallus tibetanus* at meso-scale. If the value of niche similarity indices *D* and *I* are close to 1, the niche is similar; if their value is near 0, the niche is different. The two niche similarity indices are both on the right side of 95% confidence intervals (columnar in grey) suggesting that the niches are significantly similarity (*p < .05*). By contrast, the *D* and *I* values are on the right side of 95% confidence intervals, indicating that the niches are significantly conserved (*p < .05*).


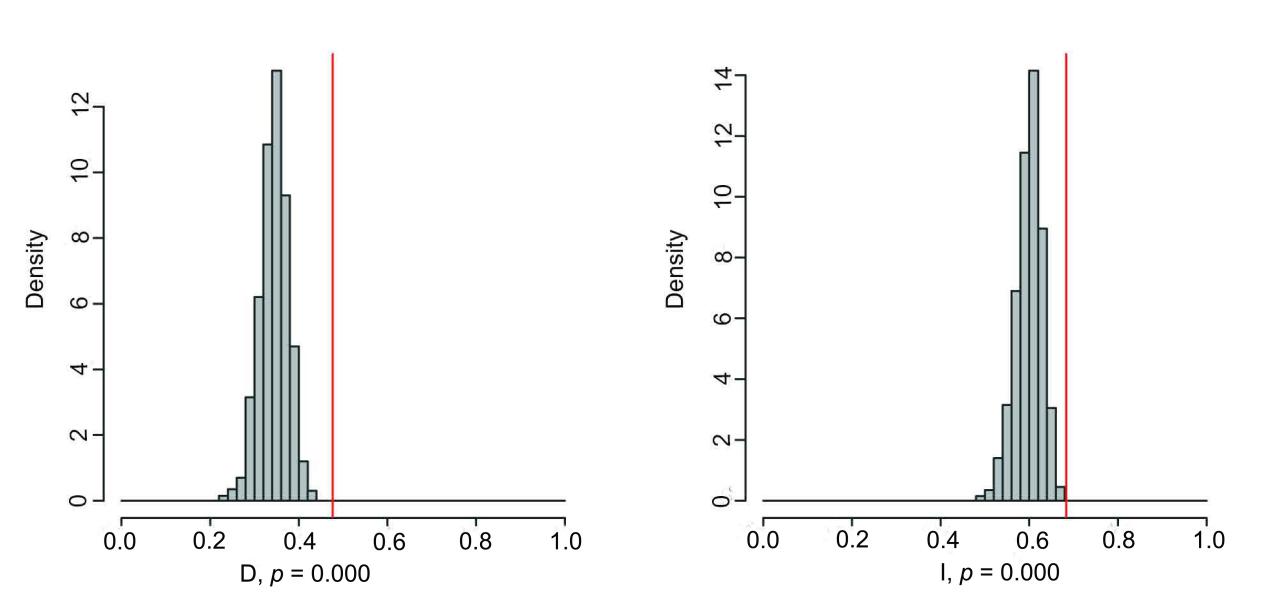


Figure S5. The results of the niche similarity test between *Lerwa lerwa* and *Tetraogallus tibetanus* at the whole-of-distribution scale. The *D* and *I* values are on the right side of 95% confidence intervals, indicating that the niches are significantly conserved (*p < .05*).
