# Supplementary material for: How do Snow Partridge (Lerwa lerwa) and Tibetan Snowcock (Tetraogallus tibetanus) coexist in sympatry under high‐elevation conditions on the Qinghai–Tibetan Plateau?
Source: Ecol Evol. 2021 Dec 8;11(24):18331–41. doi: 10.1002/ece3.8424 (PMC8717327; doi:10.1002/ece3.8424)
Supplement: Supplementary file 8 — Appendix S3 [file ECE3-11-18331-s003.docx]

**Appendix S3:**

**Table S1.** Correlation coefficients among the five environmental variables for SDMs to predict potential distribution of Tibetan Snowcock (*Tetraogallus tibetanus*) at meso-scale.

| **Variables** | **Elevation (m)** | **Aspect** | **Ridgedis (m)** | **Slope (℃)** | **Vegetation** |
| --- | --- | --- | --- | --- | --- |
| Elevation (m) | 1 |  |  |  |  |
| Aspect | ‒0.00 | 1 |  |  |  |
| Ridgedis (m) | ‒0.17 | 0.02 | 1 |  |  |
| Slope (℃) | ‒0.06 | ‒0.00 | 0.08 | 1 |  |
| Vegetation | 0.41 | ‒0.01 | ‒0.08 | ‒0.02 | 1 |

Ridgedis: the distance of a *T. tibetanus* flock to the nearest ridge line.

**Table S2.** Correlation coefficients among the eight environmental variables used for modeling and predicting the whole range distribution of Snow Partridge (*Lerwa lerwa*) and Tibetan Snowcock (*Tetraogallus tibetanus*) in the Qinghai-Tibetan Plateau.

| **Environmental variables** | **Ele (m)** | MDR (℃) | **TS** | MTDQ (℃) | **AP (mm)** | **PDM (mm)** | **PS** | **PCQ (mm)** |
| --- | --- | --- | --- | --- | --- | --- | --- | --- |
| Ele (m) | 1 |  |  |  |  |  |  |  |
| MDR (℃) | 0.20 | 1 |  |  |  |  |  |  |
| TS | 0.25 | 0.38 | 1 |  |  |  |  |  |
| MTDQ (℃) | -0.76 | -0.16 | -0.6 | 1 |  |  |  |  |
| AP (mm) | -0.38 | -0.66 | -0.71 | 0.46 | 1 |  |  |  |
| PDM (mm) | -0.16 | -0.67 | -0.72 | 0.10 | 0.44 | 1 |  |  |
| PS | -0.12 | 0.41 | -0.17 | 0.21 | -0.01 | -0.50 | 1 |  |
| PCQ (mm) | -0.08 | -0.39 | -0.08 | 0.29 | 0.27 | 0.57 | -0.43 | 1 |

Ele: Elevation;

MDR: Mean Diurnal Range (Mean of monthly (max temp – min temp));

TS: Temperature Seasonality (standard deviation *100);

MTDQ: Mean Temperature of Driest Quarter;

AP: Annual Precipitation;

PDM: Precipitation of Driest Month;

PS: Precipitation Seasonality;

PCQ: Precipitation of Coldest Quarter.

**Table S3.** The values of the 10th percentile training presence logistic threshold, the area under the receiver operating characteristic curve (AUC) and habitat suitability area for predicting the distributions of Snow Partridge (*Lerwa lerwa*) and Tibetan Snowcock (*Tetraogallus tibetanus*).

| **Species** | **Scales** | **10th percentile training presence logistic threshold** | **Average AUC value for training and testing** | **Habitat suitability area /km²** |
| --- | --- | --- | --- | --- |
| *Lerwa lerwa* | the meso-scale | 0.26 | 0.98 | 850.00 |
|  | the macro scale | 0.33 | 0.99 | 53858.00 |
| *Tetraogallus tibetanus* | the meso-scale | 0.36 | 0.98 | 956.33 |
|  | the macro scale | 0.36 | 0.96 | 906948.00 |
